# Convergent Oncostatin M and IL-31 Signaling in Chronic Pruritic Dermatoses: A Neuroimmune Janus Kinase-Signal Transducer and Activator of Transcription (JAK-STAT) Perspective

**DOI:** 10.7759/cureus.107961

**Published:** 2026-04-29

**Authors:** Michelle Tashjian, Erica K Rankin, Lily Tehrani, Marissa Ruppe, Suzanne I Riskin

**Affiliations:** 1 Department of Foundational Sciences, Nova Southeastern University Dr. Kiran C. Patel College of Osteopathic Medicine, Clearwater, USA

**Keywords:** atopic dermatitis, interleukin-31, jak-stat, nemolizumab, neuroimmune itch, oncostatin m, prurigo nodularis, pruritic dermatoses, stat3, vixarelimab

## Abstract

Chronic pruritus is a common and difficult-to-manage feature of inflammatory skin conditions. It develops through complex interactions between the skin barrier, immune system, and peripheral nervous system. Interleukin-31 (IL-31) and oncostatin M (OSM), both members of the interleukin-6 (IL-6) cytokine family, share the same receptor subunit, OSM receptor β (OSMRβ), and activate common downstream intracellular pathways, particularly the Janus kinase-signal transducer and activator of transcription (JAK-STAT) cascade. This systematic review aims to synthesize evidence in the current literature that supports IL-31 and OSM signaling across chronic pruritic dermatoses, with an emphasis on downstream JAK1-STAT3 pathways. A comprehensive search was conducted in accordance with the Preferred Reporting Items for Systematic Reviews and Meta-Analyses (PRISMA) guidelines using PubMed, Embase, Ovid MEDLINE, and the Web of Science databases. Studies evaluating OSM, IL-31, their shared receptor components (IL-31 receptor A (IL-31RA) and OSMRβ), and downstream intracellular signaling in chronic pruritic dermatoses were included. Twenty-six studies met the eligibility criteria, comprising randomized controlled trials, case-control studies, cross-sectional studies, case series, and experimental laboratory studies. Across atopic dermatitis (AD), prurigo nodularis (PN), psoriasis, cutaneous T-cell lymphoma (CTCL), dermatomyositis, and primary localized cutaneous amyloidosis (PLCA), both IL-31 and OSM contribute to chronic itch through overlapping pathways, including shared receptor architecture and activation of JAK1-STAT3 signaling. They were found to differ, however, in the way they influence neuronal activation and sensitization. IL-31 acts as a direct pruritogenic cytokine through activation of the IL-31RA/OSMRβ receptor complex on sensory neurons. In contrast, OSM may act to increase neuronal sensitivity, enhance excitability, and strengthen the response to other pruritic-inducing stimuli through the gp130/OSMRβ receptor complex. Despite these differences, both cytokines converge on the JAK1-driven STAT3 signaling pathway, ultimately contributing to skin barrier dysfunction, ongoing inflammation, and persistent neural sensitization. Blocking IL-31RA with nemolizumab or targeting OSMRβ with vixarelimab has led to clinically significant improvements in itch severity, patient-reported quality of life, and sleep disturbance. Together, these findings suggest that the shared components of the IL-31 and OSM pathways, including OSMRβ and JAK1-STAT3, provide us with a big picture framework for understanding itch pathogenesis in chronic pruritic dermatoses and may help guide therapeutic strategies in the future.

## Introduction and background

Background

Chronic pruritic dermatoses represent a broad group of inflammatory skin disorders characterized by persistent itch lasting six weeks or longer [1]. According to the International Forum for the Study of Itch (IFSI), chronic pruritus is classified based on the presence or absence of primary skin disease, with pruritic dermatoses representing itch arising directly from inflamed skin (group I) or secondary to chronic scratch lesions (group III) [2]. Common examples include atopic dermatitis (AD), prurigo nodularis (PN), psoriasis, bullous pemphigoid, and other inflammatory dermatoses in which itch is prominent and often debilitating. Proper management is warranted, as many patients experience impaired quality of life, including sleep disturbances, worsened concentration, psychological stress, and reduced daily functioning [3].

Clinically, chronic pruritic dermatoses present with variable morphologic features that evolve over time in response to sustained itch. Patients commonly exhibit erythematous lesions, hyperpigmented plaques, excoriations, or lichenification [4,5]. In AD, poorly demarcated, dry, erythematous patches tend to affect the flexural areas, whereas PN is distinguished by firm, hyperkeratotic nodules most commonly affecting the symmetric extensor surfaces. Although various presentations exist, they are unified by a shared itch-scratch cycle that contributes to a persistent state of inflammation, neural sensitization, and skin barrier disruption.

The pathogenesis of chronic pruritic dermatoses is complex and involves extensive crosstalk between the skin barrier, immune system, and peripheral nervous system [6]. Epidermal barrier disruption and chronic scratching can induce an epithelial stress response, leading keratinocytes to release "alarmin" cytokines and related mediators, such as thymic stromal lymphopoietin (TSLP), interleukin-33 (IL-33), and interleukin-25 (IL-25), periostin, and proteases (i.e., kallikreins and cathepsins) [7]. These signals ultimately promote type 2 immune responses, further exacerbating the itch-scratch cycle seen across multiple pruritic dermatoses. Notably, TSLP and IL-33 act directly on a subset of transient receptor potential ankyrin 1 (TRPA1+) sensory neurons to elicit significant pruritus [8]. Periostin, an extracellular matrix protein involved in amplifying itch, is further induced by TSLP and Th2 inflammation through activation of sensory neurons via its alpha v beta 3 (αVβ3) receptor [9]. Proteases further contribute to itch pathogenesis by exacerbating barrier dysfunction and activating protease-sensitive neuronal pathways, including protease-activated receptor 2 (PAR-2) and Mas-related G-protein-coupled receptors (MRGPR) [10].

Downstream immune activation varies by dermatosis but converges on neuroimmune pathways. In many pruritic skin diseases, type 2 cytokines, particularly interleukin-4 (IL-4), interleukin-13 (IL-13), and interleukin-31 (IL-31), promote inflammation through immune recruitment, barrier impairment, and activation of cutaneous nerve fibers [11]. Among these, IL-31, a member of the interleukin-6 (IL-6) family, has emerged as the key itch cytokine. IL-31 induces itch through binding to a heterodimeric receptor composed of IL-31 receptor A (IL-31RA) and oncostatin M receptor beta (OSMRβ) on sensory nerves in the skin, leading to downstream activation of the Janus kinase-signal transducer and activator of transcription (JAK-STAT) signaling pathway [12,13]. While IL-31 and JAK-STAT signaling have been well established as central mediators of chronic pruritus, oncostatin M (OSM) has emerged as a potential contributor to inflammatory skin disease and neuroimmune itch, given its overlap with the receptor binding of IL-31.

Oncostatin M (OSM)

OSM is a multifaceted cytokine belonging to the IL-6 family that is unique in its ability to bind to two distinct receptor complexes [14]. The type I receptor complex consists of leukemia inhibitory factor (LIF) and gp130, while the type II receptor complex consists of OSMRβ and gp130, with the latter being highly expressed by itch-selective natriuretic polypeptide B (Nppb) sensory neurons seen in chronic pruritic dermatoses [15]. OSM expression is highly upregulated in diseases associated with chronic itch, including AD, PN, and psoriasis [16]. In times of cutaneous stress, OSM is released by infiltrating immune cells, including activated T cells, monocytes, and most notably, neutrophils, the primary inducible source during inflammatory states [17]. Once OSM binds to its type II receptor complex, this induces activation of mitogen-activated protein kinase (MAPK), phosphatidylinositol 3-kinase/protein kinase B (PI3K/AKT), and associated Janus kinases (JAK), primarily JAK1 and JAK2, with downstream phosphorylation and nuclear translocation of STAT transcription factors, predominantly STAT3, and to a lesser extent STAT1 and STAT5 [18]. Rather than directly activating Nppb+ neurons, OSM sensitizes neurons by enhancing neuronal excitability and amplifying neuronal responses to pruritogenic markers such as histamine and IL-31 [19]. Through the promotion of skin hypersensitivity, OSM can worsen chronic pruritus by amplifying itch sensation, frequency, and duration.

Interleukin-31 (IL-31)

IL-31 is a pruritogenic cytokine belonging to the IL-6 family that is produced by various cell types, including CD4+ Th2 cells. It functions as a direct, non-histaminergic activator of itch in cutaneous disease. Unlike other IL-6 family cytokines, IL-31 signals independently of the common gp130 chain by binding to a heterodimeric receptor composed of IL-31RA and OSMRβ on dorsal root ganglion (DRG) sensory neurons and keratinocytes [20]. Engagement of this receptor complex can activate MAPK, PI3K/AKT, and JAK-STAT signaling, with STAT3 acting as a key transcriptional mediator for IL-31 receptor expression [21]. Activation of these pathways eventually leads to transient receptor potential vanilloid-1 (TRPV1) and TRPA1 neuronal excitation [22,23]. These activated neurons subsequently transmit itch through a specialized spinal pathway that requires neurokinin B (NKB) rather than Nppb [24].

JAK-STAT Signaling

The JAK-STAT pathway is a critical intracellular signaling axis in chronic pruritic dermatoses. This pathway responds to various extracellular cytokines, allowing it to perform several cellular responses, including immune cell regulation, epidermal homeostasis, and neuroimmune communication. The JAK family is comprised of four associated kinases (JAK1, JAK2, JAK3, and tyrosine kinase 2 (TYK2)), while the STAT family is comprised of seven proteins (STAT1, STAT2, STAT3, STAT4, STAT5A, STAT5B, and STAT6) [25]. Upon ligand binding of type I or type II cytokine receptors, intracellular JAKs become activated and phosphorylate STAT transcription factors to move to the nucleus and regulate gene expression [26]. Th2 cytokines, such as IL-4, IL-13, IL-31, IL-25, and IL-33, signaling via the JAK-STAT pathway, particularly via JAK1 and STAT3, have been associated with epidermal barrier dysfunction, chronic inflammation, and pruritus [27]. Recent studies have demonstrated the critical role of STAT3 in neurogenic pruritus, especially in IL-31-induced itch, where STAT3 expression within sensory neurons was shown to be required for both the expression of the IL-31 receptor and downstream signaling following receptor activation [28].

Rationale and aim of the study

Neuroimmune processes that directly sensitize cutaneous sensory neurons have been proposed as a mechanism behind sustained itch in chronic pruritic dermatoses. Cytokines from the IL-6 family, IL-31 and OSM, have emerged as key modulators of neuronal excitability and itch amplification. Although OSM and IL-31 signaling have each been studied as independent pathways, accumulating evidence demonstrates that they share the OSMRβ receptor subunit and converge on downstream JAK-STAT signaling pathways within itch-selective neurons. Highlighting the complementary yet nonidentical roles of IL-31 and OSM within this shared neuroimmune context allows for a more complete understanding of their respective pathways and informs therapeutic strategies for chronic pruritic dermatoses.

## Review

Materials and methods

Study Design and Eligibility Criteria

This systematic review was conducted and reported in accordance with the Preferred Reporting Items for Systematic Reviews and Meta-Analyses (PRISMA) 2020 guidelines, which are publicly available and free to use [29]. A formal protocol was not registered. Institutional review board (IRB) approval was not required as this was a secondary synthesis of published literature. Studies were eligible for inclusion if they investigated chronic pruritic dermatoses, defined as inflammatory skin diseases characterized by persistent itch for six weeks or longer. Diseases of interest included, but were not limited to, AD, PN, psoriasis, primary localized cutaneous amyloidosis (PLCA), cutaneous T-cell lymphoma (CTCL), and other related chronic inflammatory pruritic dermatoses. All included studies were required to evaluate either IL-31 or OSM or their respective receptor components, IL-31RA or OSMRβ, in the context of chronic pruritic dermatoses in humans. In addition to this requirement, studies were required to meet at least one of the following mechanistic criteria: (1) downstream intracellular signaling pathways, particularly JAK-STAT signaling; (2) experimental or translational evidence of neuroimmune mechanisms of itch; and (3) therapeutic or translational studies of itch modulation in chronic pruritic dermatoses.

Included study designs encompassed randomized controlled trials, cohort studies, case-control studies, cross-sectional studies, observational human studies, human genetic studies, tissue-based and immunohistochemical (IHC) analyses, and human dorsal root ganglion expression studies. Given the emerging and relatively novel role of OSM in chronic pruritic dermatoses, in vitro studies using human-derived cells were included to capture mechanistic evidence not yet extensively represented in clinical trials. Furthermore, mixed human-animal studies were included only when primary human data established disease relevance. Only full-text articles published in English were considered.

Studies were excluded if they were narrative reviews, systematic reviews, scoping reviews, meta-analyses, editorials, letters, commentaries, or abstract-only publications without accessible full-text. Animal-only studies and studies establishing ligand or receptor function in the absence of chronic pruritic dermatoses were excluded.

Information Sources and Search Strategy

A comprehensive systematic literature review was conducted using PubMed, Embase, Ovid MEDLINE, and the Web of Science research databases. Search strategies were designed to capture studies evaluating OSM, IL-31, their shared receptor components, and downstream JAK-STAT signaling in chronic pruritic dermatoses. Database-specific boolean operators and syntax were adapted for each platform using a combined controlled vocabulary (Medical Subject Headings (MeSH) terms) and free-text terms. Representative search terms included "oncostatin M", "interleukin-31", "OSMRβ", IL-31RA", "vixarelimab", "nemolizumab", "JAK", "STAT", "chronic pruritus", "prurigo nodularis", "atopic dermatitis", and "psoriasis". To ensure a comprehensive capture of relevant studies, the search was conducted and included all available studies up to January 2026.

Study Selection

**Figure 1:**
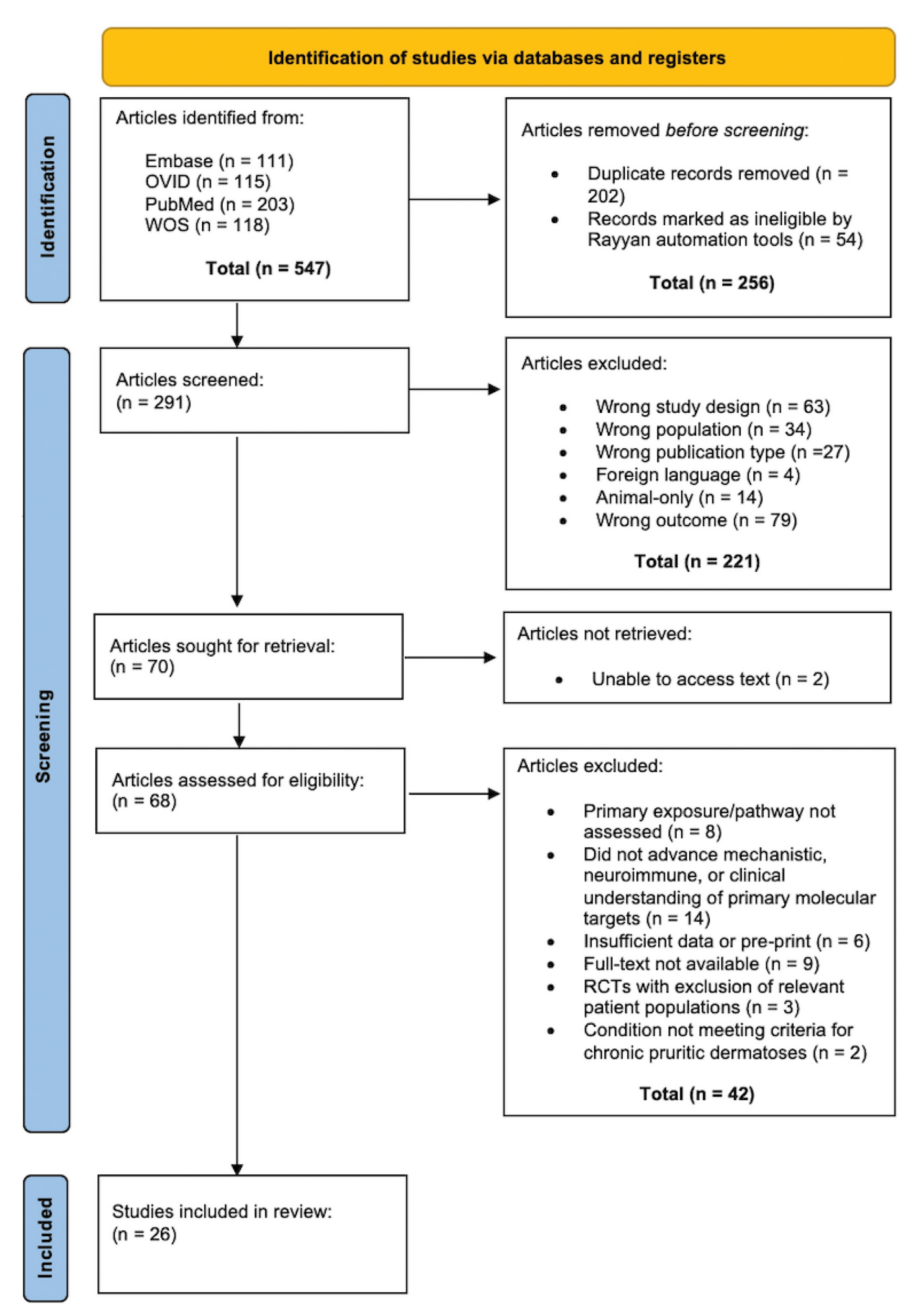
PRISMA diagram PRISMA: Preferred Reporting Items for Systematic Reviews and Meta-Analyss Source: [29]

All retrieved records were imported into the EndNote 21 application (Clarivate, London, UK), and duplicates were subsequently removed (LT). The remaining articles were uploaded to Rayyan (Rayyan Systems Inc., Cambridge, MA), where automation tools flagged ineligible studies (i.e., reviews, editorials, and commentaries). All records flagged by automation were excluded prior to tiered screening. Two reviewers (MT and ER) independently evaluated titles and abstracts (tier 1 screening), followed by full-text review (tier 2 screening) of potentially eligible studies. Disagreements at any stage of screening were resolved through discussion. If consensus could not be reached, consultation of a third reviewer (MR) was conducted. Study selection is summarized in Figure 1.

Data Extraction

For included studies, data were extracted regarding study design, study population, cutaneous disease context, sample size, and primary molecular targets. Primary outcomes extracted included itch severity and patient-reported pruritus measures (i.e., Visual Analog Scale (VAS), Peak Pruritus Numerical Rating Scale (PP-NRS), Worst Itch Numeric Rating Scale (WI-NRS), and Investigator's Global Assessment (IGA)), as well as sleep disturbance and quality of life (Dermatology Life Quality Index (DLQI)). Secondary outcomes included neuronal activation and sensitization, epidermal and immune signaling, and downstream pathway activation (JAK-STAT, MAPK/ERK, PI3K/AKT).

Risk of Bias Assessment

Methodological quality and risk of bias were assessed using the Joanna Briggs Institute (JBI) critical appraisal tools for case-control, cross-sectional, case series, and quasi-experimental studies, as well as the Cochrane risk-of-bias tool for randomized trials (RoB 2) [30,31]. Both tools are publicly available and free for academic use. Three authors (MT, LT, and MR) independently reviewed each article and categorized the risk of bias as either low, moderate, or high, based on the proportion of criteria met on the corresponding appraisal checklist. A study was considered low risk if ≥80% of items were marked "yes", moderate risk if between 50% and 79%, and high risk if <50%. Only studies where all authors assigned a score of 75% or higher were included in the final synthesis. A summary of the risk-of-bias assessment for the 26 included studies is presented in Table 1.

**Table 1:** Summary of risk-of-bias assessment for the included studies Risk of bias was assessed using the Joanna Briggs Institute (JBI) critical appraisal tools for case-control, cross-sectional, case series, and quasi-experimental studies, and the Cochrane risk-of-bias tool for randomized trials (RoB 2). The proportion of "yes" responses to checklist items corresponded to the ratings percentages. Studies scoring ≥75% were included in the final synthesis of this review. Sources: [30,31]

Study (author, year)	Study design	Appraisal tool	"Yes" (%)	Overall risk of bias
Nobbe et al. (2012) [32]	Case-control	JBI case-control checklist	75%	Moderate
Nattkemper et al. (2016) [33]	Case-control	JBI case-control checklist	90%	Low
Kim et al. (2018) [34]	Case-control	JBI case-control checklist	79%	Moderate
Boniface et al. (2007) [35]	Laboratory experimental	JBI quasi-experimental checklist	79%	Moderate
Tseng et al. (2021) [36]	Laboratory experimental	JBI quasi-experimental checklist	94%	Low
Suehiro et al. (2023) [37]	Laboratory experimental	JBI quasi-experimental checklist	94%	Low
Sonkoly et al. (2006) [38]	Case-control	JBI case-control checklist	75%	Moderate
Tey et al. (2016) [39]	Cross-sectional	JBI cross-sectional checklist	75%	Moderate
Dai et al. (2022) [40]	Laboratory experimental	JBI quasi-experimental checklist	89%	Low
Lee et al. (2012) [41]	Laboratory experimental	JBI quasi-experimental checklist	79%	Moderate
Kasraie et al. (2011) [42]	Laboratory experimental	JBI quasi-experimental checklist	75%	Moderate
Deng et al. (2023) [43]	Laboratory experimental	JBI quasi-experimental checklist	94%	Low
Wang et al. (2020) [44]	Laboratory experimental	JBI quasi-experimental checklist	75%	Moderate
Kanda et al. (2012) [45]	Cross-sectional	JBI cross-sectional checklist	79%	Moderate
Gazel et al. (2006) [46]	Laboratory experimental	JBI quasi-experimental checklist	75%	Moderate
Richards et al. (2020) [47]	Laboratory experimental	JBI quasi-experimental checklist	79%	Moderate
Hashimoto et al. (2021) [48]	Case-control	JBI case-control checklist	100%	Low
Arita et al. (2008) [49]	Case series	JBI case series checklist	94%	Low
Saeedi et al. (2014) [50]	Case series	JBI case series checklist	94%	Low
Ruzicka et al. (2017) [51]	Randomized controlled trial	Cochrane RoB 2	94%	Low
Silverberg et al. (2020) [52]	Randomized controlled trial	Cochrane RoB 2	94%	Low
Silverberg et al. (2024) [53]	Randomized controlled trial	Cochrane RoB 2	100%	Low
Ständer et al. (2025) [54]	Randomized controlled trial	Cochrane RoB 2	100%	Low
Kwatra et al. (2023) [55]	Randomized controlled trial	Cochrane RoB 2	100%	Low
Sofen et al. (2023) [56]	Randomized controlled trial	Cochrane RoB 2	94%	Low
Ständer et al. (2024) [57]	Randomized controlled trial	Cochrane RoB 2	94%	Low

Results

Overview of the Characteristics of the Included Studies

Of the 291 articles screened, a total of 26 studies were included in this review. The included studies comprised the following study designs: seven randomized controlled trials, two cross-sectional studies, five case-control studies, two case series studies, and ten experimental laboratory studies. AD, PN, PLCA, psoriasis, CTCL, dermatomyositis, and other chronic inflammatory pruritic dermatoses were among the diseases represented. Outcomes assessed include pruritus severity and sleep disturbance; expression of IL-31, OSM, IL-31RA, and OSMRβ; neuronal activation and sensitization; JAK-STAT; MAPK/ERK signaling; and clinical response to IL-31RA- or OSMRβ-targeted therapies. A summary of disease, sample size, molecular targets, findings, and limitations of included studies is shown in Table 2.

**Table 2:** Characteristics of the included studies AD=atopic dermatitis, AP-1=activator protein-1, ATAC=assay for transposase-accessible chromatin, CCL2=chemokine ligand 2, CHRNA3=cholinergic receptor nicotinic alpha 3 subunit, CTCL=cutaneous T-cell lymphoma, DLQI=Dermatology Life Quality Index, DM=dermatomyositis, DRG=dorsal root ganglia, EASI=Eczema Area and Severity Index, EdU=5-ethynyl-2′-deoxyuridine, ELISA=enzyme-linked immunosorbent assay, EQ-5d=EuroQol 5-Dimensions, FLG=filaggrin, FPLCA=familial primary localized cutaneous amyloidosis, HaCaT=human adult low calcium high temperature, HC=healthy controls, hBD-2/3=human beta-defensin-2/3, HDF=human dermal fibroblasts, HEK=human epidermal keratinocytes, HPK=human primary keratinocytes, IAP=inhibitor of apoptosis protein, IENF=intraepidermal nerve fiber, IF=immunofluorescence, IFN-gamma=interferon-gamma, IGA=Investigator's Global Assessment, IHC=immunohistochemistry, ISH=in-situ hybridization, JAK-STAT=Janus activated kinase signal transducer and activator of transcription, K1=keratinocyte 1, K10=keratinocyte 10, KRT17=keratin 17, LCE=late cornified envelope, MAPK/ERK=mitogen-activated protein kinase extracellular signal-regulated kinase, MCP-1=monocyte chemoattractant protein-1, NF-kb=nuclear factor-kappa B, NGF=nerve growth factor, NHEK=normal human epidermal keratinocytes, Nppb=natriuretic peptide B, NRS=numeric rating scale, OSM=oncostatin M, OSMRβ=oncostatin M receptor beta, PI3K/AKT=phosphoinositide 3-kinase protein kinase B, PN=prurigo nodularis, PN-NAT=prurigo nodularis nodule assessment tool, PP-NRS=Peak Pruritus Numerical Rating Scale, RHE=reconstituted human epidermis, S100A=soluble protein 100 subgroup A, SCORAD=SCORing Atopic Dermatitis, scRNA=single-cell ribonucleic acid, snRNA=single-nucleus ribonucleic acid, STAT3=signal transducer and activator of transcription 3, TLR-2=toll-like receptor 2, TrkA=tropomyosin receptor kinase A, TSLP=thymic stromal lymphopoietin, VEGF=vascular endothelial growth factor, WI-NRS=Worst Itch Numeric Rating Scale

Study (author, year)	Cutaneous disease(s) involved	Sample size	Molecular targets	Key findings	Limitations
Nobbe et al. (2012) [32]	AD (main cutaneous disease), PN, psoriasis, inverse psoriasis, alopecia areata, notalgia paresthetica, pruritus sine materia, mycosis fungoides, Sezary syndrome	Total biopsies, n=47: AD, n=5; psoriasis, n=5; inverse psoriasis, n=6; alopecia areata, n=5; PN, n=5; notalgia paresthetica, n=5; mycosis fungoides, n=5; Sezary syndrome, n=4; pruritus sine materia, n=2; HC, n=5	IL-31, IL-31RA, OSMRβ	Increased IL-31 expression was found in AD when compared with other cutaneous diseases, positively correlating with disease severity and pruritus. IL-31RA and OSMRβ immunoreactivity displayed constitutive expression in epidermal keratinocytes regardless of disease state. Lower levels of this receptor complex were detected in the dermal infiltrate.	Despite strong associations between IL-31 expression and AD, IL-31 expression was inconsistent among other dermatoses. Furthermore, these studies reflect small sample sizes, requiring further studies to assess IL-31 expression in pruritic dermatoses.
Nattkemper et al. (2016) [33]	CTCL	Human skin biopsies: CTCL, n=31; HC, n=8; itch severity stratification: moderate/severe pruritus, n=15; mild pruritus, n=8; minimal pruritus, n=8; no pruritus (HC), n=8	IL-31, IL-31RA, OSMRβ	Significant levels of IL-31, IL-31RA, and OSMRβ were found in the epidermis of patients with CTCL. IL-31 expression was also found to be increased in lymphocytic infiltrate of patients with CTCL. Patients with CTCL classified as having moderate/severe pruritus were found to have higher expression of IL-31, IL-31RA, and OSMRβ.	While associations between expression of IL-31, IL-31R, and OSMRβ were made, this study lacks an adequate amount of patients with CTCL with early stage CTCL. Additionally, there are no patients with CTCL without itch included in this study. Direct neuronal mechanisms were not assessed.
Kim et al. (2018) [34]	DM	Clinical cohort: n=191 patients total (28 men and 163 females); 73 classic DM, 118 clinically amyopathic DM; skin expression (qRT-PCR): itchy (both lesional and non-lesional); DM, n=12; HC, n=3; skin flow cytometry: itchy (lesional); DM, n=3; HC, n=4	IL-31, IL-31RA	Lesional skin samples were found to have higher mRNA levels of IL-31 and positively correlate with the VAS itch score when compared to non-lesional skin and HC. Enhanced IL-31RA gene expression was found in the lesional DM. Immunofluorescence showed increased area fraction, mean fluorescence intensity of IL-31 and mean fluorescence intensity of IL-31RA in lesional DM. Flow cytometry revealed additional sources of IL-31 expression, including CD8, CD68, CD11b, and CD11c in lesional DM. CD4+ and CD8+ cells were found in levels twofold in lesional DM compared to non-lesional and HC.	This study is unable to determine causation due to cross-sectional approach. Some of the studies relied on a small sample size. Direct neuronal mechanisms were not assessed.
Boniface et al. (2007) [35]	Psoriasis, AD	Primary human keratinocytes (n=6 NHEK donor cultures): additional human skin biopsies and T-cell experiments performed, IHC shown as representative of 4 independent experiments (donor number not specified)	OSM, type II OSM receptor, STAT3, MAPK/ERK	Human keratinocytes were found to express a functional type II OSMRβ complex on their cell surface. Stimulation of NHEK with OSM induces tyrosine phosphorylation of STAT3. Type I OSM complex was not found on the cell surface of NHEK. OSM was found to activate transcription of multiple genes involved in innate immune responses, inflammation, tissue remodeling, and angiogenesis. When added to RHE, OSM triggered hyperplasia of spinous keratinocytes, contributing to overall thickness of the tissue. Compared to healthy tissue, OSMRβ chain had increased expression levels in lesions with atopic dermatitis and psoriasis. T-cell infiltration was found to be a primary source of OSM, contributing to keratinocyte inflammation.	This study lacked clearly defined patient-level sample sizes for skin biopsies or T-cell assays. The study was largely in vitro and ex vivo design, and limited quantitative correlation.
Tseng et al. (2021) [36]	Psoriasis, AD, CTCL, inflammatory dermatoses	Human data psoriasis RNA sequencing: psoriasis, n=28; HC, n=38; AD RNA sequencing: AD, n=27; HC, n=38; CTCL RNA sequencing: CTCL, n=49; HC, n=3; animal/cellular data: mouse behavioral itch 8-18 (depending on experiment performed), calcium imaging with 236, capsaicin-responsive DRG neurons electrophysiology 8-33 neurons per condition, sensory neurons RNA; 4 pooled neuron libraries per subtype	OSMRβ, OSM, JAK1, Nppb-positive sensory neurons	OSM was found to be highly upregulated in several chronic pruritic dermatoses including psoriasis, AD, and CTCL as compared to HC. Nppb-positive itch-selective sensory neurons were found to express multiple cytokine receptors, with OSMRβ in particularly high amounts. Multi-label ISH revealed that OSMRβ and Nppb were highly co-expressed on both the mouse and human DRG. However, while most human samples demonstrated elevated OSM levels, not all did, suggesting heterogeneity in these disease groups. In RNA-sequencing skin biopsies from patients with psoriasis, AD, and CTCL, they found that amounts of OSM transcripts were significantly upregulated in all three pruritic dermatoses as compared to HC. RNA sequencing of AD and imiquimod-induced psoriasiform mouse models showed increased OSM expression as well. Sc-RNA sequencing of human CTCL and psoriatic skin showed that OSM is mainly produced by T cells and monocytes, with OSM+ cells being highly abundant in diseased versus healthy human skin. IHC of human psoriasis biopsies confirmed low CD4+/OSM+ staining in healthy skin, but strong CD4+/OSM+ immunoreactivity in lesional-psoriatic skin. For psoriasis skin biopsies, OSM-positive staining was also found to accumulate around peripheral nerve fibers. Using in vitro calcium imaging and whole-cell patch electrophysiology, they showed that OSM sensitizes Nppb-neurons rather than directly activating them through selectively potentiating histamine responses. Long-term exposure to OSM was found to significantly increase neuronal excitability as well. Through in vivo mouse models, intradermal OSM injection was found to cause delayed scratching responses by strongly potentiating histamine and leukotriene-induced itch. Conditional deletion of OSMRβ in mouse DRG was found to significantly reduce both enhanced and delayed scratch responses. Pharmacologic inhibition of OSM signaling through blocking the OSMRβ/gp130 receptor complex was shown to reduce neuronal sensitization, delayed scratch responses, and inflammation in mouse models.	Human evidence is largely observational and correlative while mouse models establish mechanistic causality of OSM in chronic pruritic disease. Not all human samples showed elevated OSM expression, while all mouse models did. Limited human sample sizes restricted generalizability. Lack of interventional human data led to uncertainty in clinical safety and efficacy of OSM and OSMRβ.
Suehiro et al. (2023) [37]	AD, psoriasis vulgaris	Human skin biopsies: AD, n=10; psoriasis, n=10; HC, n=15; serum OSM levels: AD, n=20; HC, n=20; mouse DRG and itch models, IL-31 itch assay 12 mice per group, histamine/serotonin itch assays 6 mice per group; in vitro keratinocyte assays (HEKs), n≥3, independent experiments	OSMRβ, OSM, IL-31RA, IL-31, CCL-2	OSM was highly upregulated in a majority of AD lesion samples (8 of 10 patients), and a subset of psoriasis vulgaris lesion samples (5 of 10 patients). On the other hand, OSM expression was undetectable in a majority of HC patients (14 of 15). No significant difference was seen in serum OSM of patients with AD versus HCs. In human monocytes isolated from healthy human subjects, stimulation with IL-4, GM-CSF, or both IL-4 and GM-CSF significantly increased OSM expression as compared to control samples. Stimulation of HEKs with OSM upregulated OSMRβ and downregulated IL-31RA. In patients with AD and psoriasis vulgaris, CCL-2 gene expression increased with increasing concentrations of OSM. In both fully intact and freshly excised mouse DRG, OSM stimulation upregulated OSMRβ, IL-4, and IL-13 expression while downregulating IL-31RA expression. In dispersed cultured DRGs, however, OSM stimulation increased expression of both OSMRβ and IL-31RA. In mouse skin samples, OSM stimulation did not alter receptor expression. Systemic OSM pretreatment significantly suppressed IL-31-induced itch while having no effect on histamine or serotonin-induced itch.	Although human skin samples were used to demonstrate levels of OSM expression in AD and psoriasis vulgaris, no functional neuronal experiments were performed in these human samples. This study established mechanistic causality neuronal function via murine models instead. There seemed to be a positive correlation with OSM and pruritic cutaneous disease; however, a direct correlation between OSM levels and itch severity was not established in human models. Moreover, the authors primarily measured mRNA levels of Il-31RA and other cytokine receptors instead of protein levels. This does not fully prove the amount of functional receptors on sensory neurons.
Sonkoly et al. (2006) [38]	AD, PN, psoriasis	Human skin biopsies: AD, n=35 (lesional skin, 25; non-lesional skin, 10); PN, n=5; psoriasis, n=24; HC, n=12; DRG experiment total tissues examined, n=63 (male donors, n=4; female donors, n=4)	IL-31, IL-31RA, OSMRβ	Quantitative real-time PCR was performed on IL-31 mRNA and revealed that IL-31 was significantly upregulated in pruritic AD. A subset of patients with AD (14 of 25; 56%) showed significantly increased IL-31 mRNA levels in lesional skin, which was not observed in healthy skin. Moreover, IL-31 mRNA levels in lesional AD skin showed a significant correlation with mRNA levels of CCL26, a marker whose serum levels strongly correlate with AD disease activity. Non-lesional AD skin also demonstrated significantly higher IL-31 mRNA levels compared to healthy skin. Psoriasis lesions, however, showed no difference in IL-31 mRNA expression compared to healthy skin. PN lesions had the statistically greatest increase in IL-31 expression in comparison to other chronic pruritic dermatoses and healthy skin. In a smaller subset of patients with non-lesional AD (n=6), dust mite allergen exposure induced a variable, insignificant response. However, exposure to staphylococcal superantigen rapidly and selectively induced IL-31 expression within 6 hours in all patients. Similarly, an in vitro study was conducted on peripheral blood mononuclear cells from healthy individuals (n=5) and patients with AD (n=5). Staphylococcal superantigen uniquely upregulated IL-31 expression, with significantly stronger induction in cells from patients with AD compared to HCs. Across a sample of 63 different human tissue specimens derived from 4 female and 4 male donors, IL-31RA expression was found to be highest in DRG tissue. OSMRβ was found to be similarly elevated in all tissues examined.	This study establishes strong correlations between IL-31 expression and chronic pruritic dermatoses but does not establish causality. Moreover, the authors primarily measured mRNA levels of Il-31 rather than functional protein levels. Smaller sample sizes in select cohorts, such as PN, allergen exposure, and staphylococcal antigen exposure, limited generalizability. Although IL-31RA was highly expressed in DRG tissue, this study did not demonstrate neuronal mechanisms of itch. No therapeutic interventional data was performed.
Tey et al. (2016) [39]	PLCA	Total subjects: n=40 (PLCA, n=20, with 5 patients having a family history; HC, n=20); skin biopsies/IHC (FPLCA, n=22; HC, n=22)	OSMRβ, OSM, IL-31RA, IL-31, IENFs, NGF	This study demonstrated evidence of patients with small-fiber neuropathy with macular or lichen subtypes of PLCA. Median warm detection thresholds were significantly elevated in patients with PLCA as compared to HCs. A positive correlation of warm detection thresholds and itch intensity was also reported. Although heat pain thresholds were higher at all sites of patients with PLCA compared to HCs, no statistical significance was found. IHC analysis of lesional skin showed significant reductions in intraepidermal nerve fiber density as compared to HCs. It also revealed increased epidermal expression of the IL-31 receptor complex (IL-31RA and OSMRβ) without increased IL-31 ligand, NGF, or TrkA levels in the skin or serum. The study concluded a neuropathic mechanism of itch in patients with PLCA and identified the IL-31 receptor complex as a potential therapeutic target.	Cross-sectional design study limited causality. Measurements were taken at a single time point; therefore, the study could not determine whether small-fiber neuropathy or upregulation of IL-31 receptor complex preceded itch in PLCA. The study did not directly measure neuronal excitability or signaling. Relatively small sample size restricted generalizability of the study.
Dai et al. (2022) [40]	AD	In vitro experiments with technical replicates: n=3 wells for most assays, n=4 wells for ChIP-qPCR, n=6 wells for luciferase reporter assays; findings representative of 3 independent experiments	IL-31, IL-31RA/OSMRβ, nuclear IL-33, STAT3, MAPK/ERK, FLG, K1, K10	IL-31 was found to upregulate full-length IL-33 expression in keratinocytes via activation of MAPK/ERK signaling pathways without increasing IL-33 secretion. IL-31 led to sustained activation of JAK-STAT3 signaling, which suppressed expression of FLG, K1, and K10 markers. STAT3 pharmacologic inhibition restored FLG promotor activity and expression. Nuclear IL-33 served as a transcriptional cofactor through binding to phosphorylated STAT3 and indirectly suppressing these markers as well. Knockdown of IL-33 prevented sustained nuclear localization of p-STAT3, ultimately restoring FLG, K1, and K10. IL-31 upregulation of cytokines IL-20 and IL-24 was done through a STAT3 and IL-33 dependent manner. This study suggests that increased expression of IL-33 can contribute to atopic dermatitis via skin barrier disruption.	While the study utilizes in vitro and ex vivo design, monolayer cultures and living skin equivalents may not replicate systemic interactions that occur in patients with AD. Furthermore, complete pathogenesis remains unclear as direct neuronal mechanisms were not assessed.
Lee et al. (2012) [41]	AD	Human subjects: total AD patients, n=81; extrinsic, n=65; intrinsic, n=16; HC, n=70; skin tissue samples (IHC/IF): extrinsic AD, n=4; intrinsic AD, n=4; HC, n=5; in vitro experiments: primary human keratinocytes, n=3; independent experiments per assay; murine subjects: 6 TPA mice; HC, n=6	IL-31, IL-31RA, STAT3, beta-endorphin	Activation of IL-31 in keratinocytes was found to induce calcium influx and STAT3-dependent production of beta-endorphin. IL-31, blood beta endorphins, and IL-31RA were found to be increased in patients with AD.	Potential confounders for blood beta-endorphins were not controlled for. In vivo versus in vitro concentrations of IL-31 required for beta-endorphin production vary.
Kasraie et al. (2011) [42]	AD	In vitro experiments, HPKs including foreskin-derived and hair follicle-derived keratinocytes; functional assay, n=5 independent experiments; receptor expression analyses of hair follicle HPKs: AD, n=7; HC, n=10; IL-31-induced and CCL2 secretion assays: AD, n=11; HC, n=9	IL-31, IL-31RA, OSMRβ, STAT3, TLR-2	Stimulation with ligand Pam3Cys, a synthetic bacterial lipopeptide and TLR2 ligand, resulted in upregulation of IL-31RA. IL-31 was found to activate STAT3 phosphorylation when upregulated by its receptor with Pam3Cys or IFN-gamma in both foreskin-derived and hair follicle-derived keratinocytes. Pam3Cys-induced upregulation of IL-31 resulted in increased levels of MCP-1/CCL2, VEGF, and CCL22. Pam3Cys upregulation of IL-31RA in keratinocytes from AD were found in lower expressions compared to HC. Decreased expression of TLR-2 was found on immunohistological staining of punch biopsies from patients with AD.	This study is largely in vitro, relying on stimulated keratinocytes rather than direct functional testing in vivo. Further limitations are imposed due to the small sample size. Punch biopsy analyses are qualitative versus quantitative based off IHC.
Deng et al. (2023) [43]	Moderate-severe PN	Total patients, n=38 (mean age: 55.8 years; 22 women, 16 men); nemolizumab responders, n=19; placebo responders, n=19	IL-31RA blockade	193 proteins were found to be differently expressed after 12-week treatment with nemolizumab compared to placebo. Those who received nemolizumab showed downregulation in inflammatory signaling and pruritus and/or neural dysregulation. Among the pathways downregulated were acute phase response proteins, VEGF, and STAT3.	This study was performed in patients originally from phase 2 clinical trials for PN, with potential to inflate type I errors. Moreover, the relatively small cohort (n=38) and short-term duration of the study pose a limitation to study design.
Wang et al. (2020) [44]	Psoriasis vulgaris	In vitro human HaCaT keratinocytes: n=3 independent experiments per assay	OSMRβ, OSM, MAPK/ERK, STAT3, Livin	Livin, a member of the IAP family, is upregulated in psoriatic lesional skin, and higher levels correlate with worse disease severity. OSM was identified as an upstream enhancer of Livin in psoriatic keratinocytes. OSM stimulation of HaCaT keratinocytes led to a time-dependent increase in Livin mRNA and protein expression. OSM led to induction of transcription at 6 hours, with peak levels at 12 hours, while protein expression rose within 2 hours. Within 24 hours, Livin levels declined yet remained elevated in comparison to unstimulated controls. OSM-treated HaCaT keratinocytes demonstrated enhanced keratinocyte survival under oxidative stress conditions through induction of both Livin and EdU, a marker of cell proliferation. Rapid activation of STAT3 and ERK signaling pathways was also seen in OSM-treated keratinocytes. Pharmacologic inhibition of STAT3 via cryptotanshinone, and ERK via U0126 significantly reduced OSM-induced Livin expression. In this study, Livin-mediated survival was found to depend on ERK activation, but not STAT3 activation.	In vitro models using healthy donor keratinocytes rather than lesional keratinocytes. Although this study establishes mechanistic causality under controlled conditions, findings may not fully translate into human disease and may not capture interactions that occur in vivo. Functional conclusions are based on Livin overexpression rather than loss-of-function approaches, leading to possible bias.
Kandaet al. (2012) [45]	AD	Human subjects: AD, n=26 (mean age: 38.3 ± 13.0; 16 men, 10 women); HC, n=27 (mean age: 37.3 ± 6.3; 19 men, 8 women); in vitro keratinocytes, human neonatal foreskin, isolated CD3+ T cells: AD, n=2; normal donors, n=2	hBD-2, hBD-3, IL-22, OSM, IL-31, IL-25, TSLP, STAT3	Serum hBD-2 was found to directly correlate to AD disease activity, while serum hBD-3 had moderate increases that were not statistically significant when compared to HC. Serum hBD-2, IL-22, and OSM were higher in patients with AD compared to HCs, whereas serum IL-31, IL-25, TNF-a, and IL-10 levels were significantly lower. Serum hBD-3, TSLP, IL-4, and IL-13 levels were not significantly different from HC. IL-22 and OSM were found to strongly increase hBD-2 secretion, while TSLP, IL-13, and IL-4 decreased secretion. Serum hBD-3 secretion exhibited a moderate increase when stimulated by IL-22 and OSM. OSM increased hBD-2/hBD-3 production primarily through STAT3 and ERK signaling, whereas IL22 increased hBD-2/hBD-3 production through STAT3 and p38 MAPK signaling. STAT3 inhibition significantly reduced IL-22 and OSM induction of hBD-2/hBD-3. Higher hBD-2 levels in the serum increased IL-22 and OSM production in T cells. Higher hBD-3 in the serum increased IL-31, IL-4, and IL-13 production in T-cells.	Despite finding increased serum hBD-2 levels in AD, this study is limited by a small cohort size. Additionally, the mechanistic T-cell experiments are based on a small donor pool (4 separate experiments using T cells from 2 normal donors and 2 patients with AD). Biomarker outcomes were based off serum measurements instead of direct paired lesional skin quantification in the same cohort, leading to indirect findings. Finally, in vitro concentrations were significantly higher than serum concentrations, limiting in vivo assessments.
Gazel et al. (2006) [46]	Psoriasis	In vitro human skin equivalents from multiple donors (number not specified); approximately 12,000 genes profiled per condition within 10 arrays across 5 time points + controls; qRT-PCR RNA, n=3 epidermal tissues, western blot protein from 3 epidermal tissues	OSM, type II OSM receptor, JAK-STAT, S100A family, KRT17, epidermal differentiation markers (FLG, loricrin, involucrin, LCE genes)	OSM induces a distinct subset of psoriasis-associated transcription factors in reconstructed epidermis. Among the genes induced, strong induction of S100A proteins and KRT17 were found. On the other hand, markers such as loricrin, FLG, and involucrin were suppressed. Within human skin equivalents, OSM was also found to trigger a biphasic response of induction with subsequent suppression in a subset of IFN-regulated genes including STAT1. OSM consistently was found to induce STAT3 expression within these reconstituted epidermis models.	This in vitro model used healthy donor keratinocytes rather than lesional keratinocytes, limiting exploration of the immune cell components behind psoriasis pathology. Additionally, the biological replicates for microarray conditions were not clearly reported.
Richards et al. (2020) [47]	Chronic inflammatory pruritic dermatoses	In vitro (HEK + HDF): ELISA, n=4 wells per condition; RNA, n=3 wells per condition; neutralization/antibodies, n=4 wells per condition	OSM, OSMRβ, CCL2, STAT3, STAT1	OSM was found to regulate expression of pro-inflammatory chemokine MCP-1/CCL2 by HEK and HDF cells, suggesting that OSM via OSMRβ contributes to inflammatory conditions. In HEKs, OSM stimulation was found to significantly induce STAT1 and STAT3 phosphorylation, as well as increase MCP-1 production when combined with IL-4 or IL-13. KPL-716, an OSMRβ inhibitor, was found to significantly reduce MCP-1 response to OSM stimulation in HEKs and HDFs.	Further studies are needed to establish the molecular pathogenesis to determine if the MCP-1 gene promoter has response elements for STAT1, STAT3, and STAT6 transcription factors. Additionally, the correlation between OSMRβ and gp130 protein levels accompanying increased OSM sensitivity has yet to be established.
Hashimoto et al. (2021) [48]	PN	Human skin biopsy specimens: PN, n=25; HC, n=10; dermal itch correlation subset: n=8 (punch biopsies with recorded NRS)	IL-31, OSM, IL-31RA, OSMRβ	Dermal expression of IL-31, IL-31RA, and OSM cells positively correlated with severe itch intensity. Dermal OSMRβ cells were increased in PN lesions but did not significantly affect itch severity. Within the epidermis of PN lesions, OSMRβ cells were lower in the epidermis and inversely correlated with itch. Overall, dermal OSM expression had a significant association with itch intensity in PN.	While itch intensity in PN was found to correlate with increased dermal levels of certain cytokines, larger-scale studies to confirm these findings are still needed. Furthermore, limitations are imposed by the small sample size of patients with PN and lack of control for disease variability between patients with PN. Finally, protein expression was assessed only by IF, leaving multiple other cellular sources unassessed. Funding and conflict of interest by Kiniksa may also limit this study’s findings.
Arita et al. (2008) [49]	Autosomal-dominant FPLCA	21 affected individuals from 3 unrelated families: family 1 (Brazilian): 16 affected, individuals across four generations; family 2 (British): 3 affected individuals; family 3 (South African): 2 affected individuals	Recombinant human OSM, IL-6, IL-31	Heterozygous point mutation in OSMRβ resulting in amino acid change of isoleucine to threonine at amino acid 691 was found in all affected individuals in family 1. A heterozygous missense mutation was common to all affected individuals in families 2 and 3, suggesting common ancestors. Mutations in FPLCA families were found to occur within the extracellular fibronectin type III-like domains of OSMRβ. In FPLCA, levels of pSTATs, pErk, and pAkt were reduced 65%-95% after OSM stimulation, suggesting receptor dysfunction. Phosphorylation was not observed following IL-31 stimulation in the FPLCA group. Additionally, phosphorylation was not altered following IL-6 stimulation in FPLCA keratinocytes.	This study established mutations common to individuals affected by FPCLA across different families; however, the study is unable to establish causality. Additionally, the study was limited by the small sample size.
Saeedi et al. (2014) [50]	FPLCA	FPLCA, n=4 patients from a Kurdish family in 3 consecutive generations (father, two daughters, and one granddaughter); HC, n=100 ethnically matched patients	OSMRβ, OSM JAK-STAT, MAPK, PI3K/AKT	This study identified a new mutation in the OSMRβ observed in PLCA affected family members. A C/T substitution was discovered at position 613, resulting in a leucine to serine substitution. This substitution sits in the extracellular fibronectin type III domain of the OSMRβ receptor. HCs lacked this substitution.	This study is limited due to the restrictions imposed on findings from one singular family. Further studies are needed to understand the exact molecular pathogenesis as direct neuronal mechanisms were not assessed.
Ruzicka et al. (2017) [51]	AD	Total patients, n=264; nemolizumab, n=211; placebo, n=53; 216 patients completed the 12-week treatment period	IL-31, IL-31RA	This phase 2 clinical trial was aimed at understanding the efficacy of IL-31R antagonist, nemolizumab, in patients with moderate-to-severe AD. At all doses, nemolizumab showed clinical improvements in pruritic symptoms at the end of 12 weeks when compared with baseline. A clear dose-response pattern was also recorded. At week 12, EASI scores in the 0.5 mg/kg and 2.0 mg/kg doses of nemolizumab were significantly improved compared to placebo. EASI findings for the 0.1 mg/kg group were insignificant at this time point. SCORAD outcomes improved significantly in all doses of nemolizumab when compared to placebo. Sleep VAS scores were significantly improved in all nemolizumab groups as compared to placebo.	This study experienced a high dropout rate (18%) potentially skewing results. Additional limitations are due to a relatively small sample size and short duration of experiment. This study lacks the ability to establish adverse events. Direct neuronal mechanisms were not assessed.
Silverberg et al. (2020) [52]	Moderate-severe AD	Total patients, n=226 (mean age: 39.3; 115 males, 111 females): 10 mg nemolizumab, n=55; 30 mg nemolizumab, n=57; 90 mg nemolizumab, n=57; placebo, n=57	IL-31, IL-31RA	All nemolizumab doses showed significantly greater improvement in mean EASI scores compared to placebo. On week 24, the greatest difference from baseline was observed in the 30 mg nemolizumab dose, with greatest statistical significance observed at week 16. The 30 mg nemolizumab dose in particular demonstrated the strongest efficacy in this phase 2b clinical trial. At this dose, the greatest reductions in EASI scores were seen in weeks 16 and 24. The 30 mg nemolizumab dose also achieved the highest responder rates for EASI50, EASI75, and EASI90, significantly outperforming placebo. While all doses of nemolizumab significantly improved PP-NRS compared to placebo, the 30 mg dose of nemolizumab achieved the most pronounced effect by 1 week, and the largest magnitude of itch reduction by week 24. IGA success with the 30 mg nemolizumab group was the highest, significantly surpassing both placebo and other nemolizumab doses during weeks 12-24. On week 24, all 3 doses of nemolizumab significantly improved SCORAD scores compared to placebo, with the 30 mg nemolizumab group achieving the greatest difference. Across all doses of nemolizumab, especially in the 30 mg group, mean reductions in DLQI and EQ-5D demonstrated significant improvement in sleep disturbance and quality of life.	The placebo-controlled portion of this phase 2b clinical trial lasted 24 weeks, which restricted long-term efficacy and durability of medication response. While multiple doses were being tested, the small sample sizes in each group limited efficacy and generalizability of the study. Clinical efficacy of IL-31 blockade was established, but direct neuronal mechanisms were not assessed.
Silverberg et al. (2024) [53]	Moderate-severe AD	Total patients, n=1,728 (ARCADIA I, n=941; ARCADIA II, n=787); nemolizumab plus topical therapy total patients, n=1,142 (ARCADIA I, n=620; ARCADIA II, n=522); placebo plus topical therapy total patients, n=586 (ARCADIA I, n=321; ARCADIA II, n=265)	IL-31, IL-31RA	The majority of participants in both trials (ARCADIA I and ARCADIA II) had baseline IGA scores of 3 and mean PP-NRS scores ranging from 7 to 7.2. In both trials, by week 16, there were significantly greater reductions in pruritus and sleep disturbance seen with the nemolizumab + topical therapy group versus the placebo + topical therapy group. These findings were seen as early as week 1 of the study. ARCADIA I: IGA success scores of 0 or 1 were observed in 36% of the nemolizumab + topical therapy group versus 25% of the placebo + topical therapy group. An EASI-75 response occurred in 44% of the nemolizumab + topical therapy group versus 29% of the placebo + topical therapy group. ARCADIA II: IGA success scores of 0 or 1 were observed in 38% of the nemolizumab + topical therapy group versus 26% of the placebo + topical therapy group. An EASI-75 response occurred in 42% of the nemolizumab + topical therapy group versus 30% of the placebo + topical therapy group.	The placebo-controlled portion of these phase 3 clinical trials lasted 16 weeks, which restricted long-term efficacy and durability of medication response. Although a large sample size was used, a majority of the participants were white, therefore underrepresenting other races and ethnicities, and restricting generalizability. The use of concomitant topical therapy also limited the ability to assess IL-31 blockade alone. The weights of the topical therapy tubes were not measured, which could also interfere with the results. Direct neuronal mechanisms were not assessed.
Ständer et al. (2025) [54]	Moderate-severe PN	Total patients randomized, n=286 (mean age: 57.5 ± 13.0; 120 males, 166 females): nemolizumab, n=190; placebo, n=96	IL-31, IL-31RA	Nemolizumab produced clinically meaningful itch reductions as early as week 4, with 41.1% of patients in the nemolizumab group achieving a significant itch response compared to 6.3% of patients in placebo. By week 16, a significantly higher proportion of patients in the nemolizumab group achieved ≥4-point improvement in PP-NRS compared to placebo (58.4% versus 16.7%). Itch outcomes were sustained through week 24. Higher rates of IGA success were reported in the nemolizumab group compared to placebo (26.3% versus 7.3%), indicating improved skin disease severity.	The study population was unrepresentative of all patients with PN as it was predominantly conducted on white patients and excluded patients with uncontrolled comorbidities. This OLYMPIA 1 phase 3 clinical trial had a duration of 24-week treatment and 8-week follow-up, which was a short time period given the chronicity of PN. Studies with longer duration are necessary to support long-term use in patients with PN. No head-to-head comparisons with other biologics such as dupilumab were performed in this study. The clinical efficacy of IL-31 blockade was established, but direct neuronal mechanisms were not assessed.
Kwatra et al. (2023) [55]	Moderate-severe PN	Total patients, n=274: nemolizumab, n=183; placebo, n=91; 262 patients completed 16-week treatment period, 257 entered long-term extension study	IL-31RA	IL-31 signaling blockade with nemolizumab resulted in a 56.3% decrease in itch intensity compared to a 20.9% reduction in placebo group. IGA success was achieved by week 4 in the nemolizumab group and sustained through week 16. Improvements in sleep disturbance, DLQI, EQ-5D, and symptoms of anxiety and depression were seen in the nemolizumab group as compared to placebo counterparts.	This study excludes patients with inadequately controlled systemic conditions. Most participants of the trial are white, leading to a lack of generalizability to the public.
Sofen et al. (2023) [56]	PN	Total patients, n=50: vixarelimab, n=23; placebo, n=26	OSMRβ, OSM	Compared to baseline, WI-NRS scores at week 8 were significantly reduced in the vixarelimab group (-50.6%) compared to the placebo group (-29.4%). Highly statistical differences were seen as early as week 3 and were sustained through week 8. By week 8, 52.2% of the vixarelimab group achieved a ≥4-point reduction in WI-NRS compared to 30.8% of the placebo group. Highly statistical differences were observed in weeks 3, 4, and 5. Large-magnitude reductions (i.e., 6, 7, and ≥8-point) from baseline were also observed in the vixarelimab group as early as week 3. Higher rates of an IGA score of 0 or 1 were also reported in the vixarelimab group (30.4%) versus placebo group (7/7%) by week 8, indicating improved skin disease severity. Higher reductions from baseline in PN-NAT scores were observed in the vixarelimab group almost every week. DLQI and sleep loss scores were significantly improved as early as week 2 and sustained through week 8.	The placebo-controlled portion of this phase 2a clinical trial lasted 8 weeks, which restricted long-term efficacy and durability of medication response. The relatively small sample size as well as exclusion of patients with AD as a comorbidity restricted the generalizability of this study. Some assessment tools used, including the PN-NAT, are new and not yet validated in PN. The clinical efficacy of OSMRβ blockade was established, but direct neuronal mechanisms were not assessed.
Ständer et al. (2024) [57]	Moderate-severe PN	Total patients, n=190 (mean age: 55.4 ± 13.8; 75 males, 114 females); receiving ≥1 dose: n=189; vixarelimab total, n=141: high dose (540 mg), n=47; medium dose (360 mg), n=47; low dose (120 mg), n=47; placebo, n=48	OSMRβ, OSM	Vixarelimab significantly reduced mean WI-NRS scores at week 16 in high-dose (-56.2%), medium-dose (-51.0%), and low-dose (-33.0%) as compared to placebo (-14.5%). Reductions in WI-NRS were seen as early as week 1 and sustained throughout the phase 2b trial. Clinically meaningful ≥4-point reductions in WI-NRS were also proportionally higher in the high-dose (66.0%), medium-dose (61.7%), and low-dose (29.8%) vixarelimab as compared to placebo (16.7%). IGA scores of 0 or 1 were subsequently reported across all doses of vixarelimab as compared to placebo, indicating improved skin disease severity. Through dual inhibition of IL-31 and OSM pathways, vixarelimab demonstrated similar efficacy in clinically meaningful itch outcomes in PN as nemolizumab did in the OLYMPIA trials.	The placebo-controlled portion of this phase 2b clinical trial lasted 16 weeks, which restricted long-term efficacy and durability of medication response. An open-label extension was created; however, there is a lack of a placebo comparator group. The clinical efficacy of OSMRβ, blockade was established, but direct neuronal mechanisms were not assessed.

Cellular Sources and Targets of IL-31 and OSM in Chronic Pruritic Skin

Chronic pruritic dermatoses are marked by dense immune cell infiltration in lesional skin. Within this microenvironment, cytokines, such as IL-31 and OSM, play a critical role in itch pathogenesis. Across the included literature, it has become evident that these two cytokines serve as upstream mediators linking cutaneous inflammation to neuronal sensory signals. Describing their cellular sources and targets within the skin, therefore, provides critical context before diving into neuroimmune signaling mechanisms.

IHC analyses revealed increased IL-31 protein levels within the dermal inflammatory infiltrates of patients with AD as compared to healthy controls [32]. On the other hand, regardless of disease state, the IL-31 receptor complex, IL-31RA and OSMRβ, was found to be broadly expressed in epidermal keratinocytes and present in low levels within these dermal infiltrates. These findings suggest a ligand-driven mechanism in AD, where increased IL-31 production within dermal immune infiltrates, such as CD4⁺ Th2 cells, can act on its constitutively expressed receptors present on other cells to elucidate an itch effect.

In another study conducted on CTCL, IL-31 protein expression was found to be significantly increased in both epidermis and dermal lymphocytic infiltrates [33]. All subjects, regardless of disease state, had IL-31 expression limited to the lymphocytic infiltrate, but higher amounts of IL-31 detected in these sources correlated with worse itch severity. Unlike the AD findings previously described, IL-31RA and OSMRβ were found to be significantly upregulated in the epidermal keratinocytes of patients with CTCL. These findings suggest that both ligand production and receptor amplification within the epidermis contribute to worsened itch outcomes. Immunofluorescence (IF) evaluating lesional skin samples of patients with dermatomyositis (DM) revealed that CD4+ T-cells were the predominant source of IL-31 production [34]. Flow cytometry models revealed CD8+ T-cells, dendritic cells, monocytes, and cells of myeloid lineage as potential contributors.

OSM production within lesional skin has also been found to localize infiltrating immune cells. Protein-level analyses investigating inflammation and pruritus in AD and psoriasis found that T-cells isolated from lesional skin produced higher levels of OSM than T-cells from the peripheral blood, therefore identifying skin-infiltrative T-cells as a primary source of OSM [35]. Compared to healthy tissue, they also found that the type II OSM receptor, composed of gp130 and OSMRβ, had increased protein levels in the lesional skin of both conditions. Transcriptomic profiling of inflammatory disorders also found significantly increased OSM single-cell RNA transcripts within T-cells and monocytes in diseased skin versus healthy controls [36].

Neuroimmune Framework of IL-31 and OSM in Chronic Itch

Although IL-31 and OSM share the OSMRβ subunit, they play different, yet complementary roles as mediators of neuroimmune itch. An in vitro model of human epidermal keratinocytes (HEKs) demonstrated how OSM stimulation led to upregulation of OSMRβ while downregulating IL-31RA [37]. To test this on a neuronal level, systemic OSM administration in mice produced similar effects in intact DRG.

Early human tissue analyses demonstrated upregulation in IL-31 mRNA levels in AD and PN [38]. Concurrent mapping of IL-31RA revealed that this receptor was most highly expressed in the DRG as compared to other peripheral tissues. Together, these findings positioned IL-31 as a cytokine with direct access to sensory neuronal pathways implicated in itch transmission.

While IL-31 acts as a direct pruritogenic signal, OSM has been found to act as a modulator of neuronal itch sensitization. Mechanistic studies examining multiple inflammatory dermatoses, including AD, psoriasis, and CTCL, demonstrated that OSM is upregulated in these conditions and preferentially targets itch-selective Nppb-positive sensory neurons [36]. In vitro and in vivo models demonstrated how OSM sensitizes Nppb neurons to classic pruritogens such as histamine, resulting in delayed and amplified scratch responses. Pharmacologic blockade of OSM signaling was found to reduce these intense, delayed itch behaviors.

In an IHC analysis of patients with PLCA, lesional skin demonstrated significant reductions in intraepidermal nerve fiber density and elevated warm detection thresholds, which positively correlated with worse itch outcomes [39]. Increased epidermal expression of the IL-31 receptor complex (IL-31RA and OSMRβ) without increased IL-31 ligand levels was also observed in these patients. This study demonstrated how dysregulated IL-31RA and OSMRβ signaling at the neuronal level can worsen chronic itch burden.

Convergent JAK-STAT Signaling as a Downstream Neuroimmune Hub 

IL-31 and OSM activate overlapping intracellular cascades centered on the JAK-STAT pathway. Across both cutaneous and neuronal contexts, STAT3 emerges as a recurring hub for cutaneous inflammation, neuronal sensitization, and pruritus. Additional engagement of MAPK/ERK and PI3K/AKT signaling has also been reported in different cell types and contexts.

IL-31-driven signaling: Multiple studies have demonstrated STAT3 as a central downstream mediator of IL-31 signaling. In studies investigating AD, Dai et al. found that IL-31 stimulation in normal HEK (NHEK) inhibited filaggrin (FLG), keratin (K) 1, and K10 through sustained activation of the JAK1-STAT3 signaling pathway [40]. Moreover, STAT3 inactivation in both in vitro and ex vivo experiments led to increased FLG protein levels in IL-31-treated models [40]. Another study investigating AD found a positive correlation between IL-31 and beta-endorphin levels. IL-31RA activation in keratinocytes was found to increase blood beta-endorphin levels through inducing calcium influx and STAT3 activation [41]. Inhibition of STAT3 reduced the increase of these beta-endorphins in patients with AD, further supporting STAT3 as a critical downstream mediator of IL-31 activity. Not only was IL-31 induction of STAT3 witnessed across several cells and tissues, but in primary human keratinocytes (PHK), further amplification of this signaling was reported following toll-like receptor 2 (TLR2) activation with Pam3Cys or interferon-gamma (IFN -γ) [42]. In a biomarker analysis of patients with moderate-to-severe PN treated with nemolizumab, an IL-31RA antagonist, downregulation of inflammatory pathways, including JAK1-STAT3 signaling, was reported in treatment responders as compared to placebo [43].

OSM-driven signaling: OSM binding to its OSMRβ/gp130 receptor complex has also been found to cause downstream activation of JAK-STAT pathways, with prominent engagement of STAT3. In HEK models undergoing oxidative stress, OSM stimulation promoted keratinocyte survival and proliferation through rapid activation of STAT3 and ERK signaling pathways [44]. Pharmacologic inhibition of STAT3 and ERK significantly reduced OSM-induced expression of survival-associated genes such as Livin. Blood serum analyses of human β-defensins (hBD) as a biomarker of AD severity also demonstrated the downstream signaling effects of OSM [45]. OSM signaling was found to increase serum hBD-2 through STAT3 and ERK signaling, which generates feedback to T-cells to produce greater amounts of OSM cytokine. Transcriptomic profiling of reconstituted human epidermis suggested a functional crosstalk between OSM and IFN, where OSM treatment induced STAT3 signaling while suppressing IFN-regulated pathways, including STAT1 [46]. These findings demonstrated STAT3 as a key downstream mediator of OSM signaling. On the other hand, OSM induction in HEK and human dermal fibroblasts (HDF) induced both STAT1 and STAT3 signaling [47].

Disease-Specific Findings Within the Included Studies

Atopic dermatitis: Across multiple studies, AD has been consistently supported by IL-31 pathway evidence with complementary but more variable findings for OSM. Patients with AD demonstrated increased IL-31 expression when compared to HCs and also demonstrated elevated mRNA levels in both lesional and non-lesional skin [38]. IHC analyses of multiple pruritic dermatoses, including AD, PN, psoriasis, and CTCL, found the highest level of IL-31 protein in AD lesions [32]. In keratinocyte-based AD models, IL-31 induction of nuclear IL-33 and phosphorylation of STAT3 was found to suppress key epidermal differentiation markers in AD, such as FLG, K1, and K10 [40]. IL-33 knockdown models and pharmacologic inhibition of STAT3 have demonstrated restoration of these markers in AD. AD-associated studies have also described higher serum OSM in atopic patients. Through in vitro keratinocyte models, stimulation of OSM has been shown to increase serum hBD-2, which in turn has increased OSM production from activated T-cells, and therefore established a cytokine amplification loop of perpetual inflammation in AD [45].

Prurigo nodularis: Compared to other chronic pruritic dermatoses, PN has exhibited particularly strong enrichment of both IL-31 and OSM signaling. Immunofluorescence analyses of PN lesions revealed increased dermal expression of IL-31- and OSM-positive cells that directly correlated with itch intensity [48]. Dermal OSMRβ-positive cells were significantly greater in PN lesions compared with HCs, while epidermal OSMRβ-positive cells were reduced. Although dermal receptor density did not significantly correlate with itch outcomes, epidermal expression was inversely correlated with itch. The differential distribution of OSM and OSMRβ across the dermal and epidermal layers suggested layer-specific compartmentalization of OSM signaling in PN. Furthermore, in moderate-to-severe PN, plasma proteomic profiling following nemolizumab treatment showed significant downregulation in inflammatory signaling, pruritus, synaptogenesis, and overall neural dysregulation [43].

Primary localized cutaneous amyloidosis: Dominant and recessive OSMRβ mutations identified in PLCA provide direct human evidence linking impaired OSMRβ signaling to severe chronic pruritus. In autosomal-dominant PLCA, Arita et al. and Saeedi et al. identified heterozygous missense mutations within the extracellular fibronectin type III-like domains of OSMRβ, regions critical for receptor dimerization and signal transduction [49,50]. Following stimulation with OSM and IL-31, activation of downstream JAK-STAT, MAPK/ERK, and PI3K/AKT pathways was markedly reduced. On the other hand, downstream signaling pathways were preserved with IL-6 administration, supporting OSMRβ as a shared subunit critical to chronic pruritus.

Psoriasis: In psoriasis, the role of OSM in chronic itch was connected closer to epidermal hyperproliferation and inflammatory remodeling rather than a Th2-driven process. OSM was identified as an upstream enhancer of Livin, a member of the inhibitor of apoptosis (IAP) family known to be elevated in the lesional skin of psoriasis vulgaris patients. An in vitro model treating human adult low-calcium high-temperature (HaCaT) keratinocytes with OSM demonstrated enhanced keratinocyte proliferation under exogenous hydrogen peroxide conditions [44]. Despite the induction of oxidative stress, OSM was able to upregulate Livin and 5-dihydro-2'-deoxyuridine (EdU), a marker of cell proliferation, to promote keratinocyte survival.

Reconstructed human epidermis models have further demonstrated the ability of OSM to increase keratinocyte proliferation and survival through induction of a distinct subset of psoriasis-associated transcription factors such as keratin 17 (KRT17) and the S100A family [46]. Tseng et al. analyzed RNA-sequencing data from skin biopsies of psoriasis patients and found significant upregulation of OSM transcripts in this population as compared to controls [36]. They also found high levels of OSM expression on IHC staining with accumulation around peripheral nerve fibers.

Other pruritic dermatoses: Pruritus in CTCL has been associated with the upregulation of IL-31 pathway components. Both epidermal and dermal expression of IL-31 and its receptor complex, IL-31RA and OSMRβ, were found to be significantly elevated in CTCL as compared to HCs [33]. Highest expression levels of IL-31 were observed in patients with moderate-to-severe pruritus; however, these cytokine levels had no effect on tumor stage. In regard to pruritus outcomes, quantitative amounts of IL-31 were directly correlated with itch intensity.

Lesional skin samples of patients with DM were found to have higher mRNA levels of IL-31 and IL-31RA compared to non-lesional skin samples and HCs [34]. Levels of IL-31 expression and cutaneous severity of DM lesions positively correlated with itch intensity. Flow cytometry revealed multiple immune cell populations contributing to IL-31 mRNA expression within lesional tissue.

Therapeutic Interventions Targeting the IL-31 and OSM Signaling

Clinical trials targeting IL-31 and OSM signaling have demonstrated great safety and efficacy in reducing itch outcomes in addition to quality of life and sleep disturbance measures. Therapeutic inhibition of IL-31RA with nemoluzimab, or of OSMRβ with vixarelimab, has emerged as a promising class of antipruritic biologics.

IL-31 targeted therapies: Many of the nemolizumab clinical trials have been conducted on patients with AD and PN. By week four in the phase 2 randomized trial conducted by Ruzicka et al., nemolizumab significantly and rapidly improved pruritus outcomes across all nemolizumab groups in a dose-dependent manner [51]. By week 12, eczema area severity index (EASI), scoring atopic dermatitis (SCORAD), and sleep VAS scores significantly improved at higher doses of nemolizumab when compared to placebo. A subsequent phase 2b trial by Silverberg et al. demonstrated significant improvements in all doses of nemolizumab, but particularly found the highest statistical and clinical efficacy in the 30-mg nemolizumab group [52]. Peak Pruritus Numerical Rating Scale (PP-NRS) reductions were seen as early as week one and sustained through week 24 in the 30-mg nemolizumab group. EASI scores, lesion clearance, sleep disturbance, and quality-of-life measures all significantly improved at this dose as well. Larger phase 3 trials (ARCADIA I and II) evaluating nemolizumab plus topical therapy versus placebo plus topical therapy further validated these findings [53]. Significant reductions in pruritus, disease severity, and sleep disturbance were reported in the nemolizumab group compared to placebo.

In PN, nemolizumab produced similar effects on measures of itch reduction and skin disease burden. In the OLYMPIA 1, phase 3 clinical trials, nemolizumab produced clinically meaningful itch reductions and improvements in peak pruritus scores from weeks 4 through week 24 [54]. Improvements in sleep disturbance, lesion clearance, and quality of life were also observed. Similarly, in the OLYMPIA 2 phase 3 clinical trials, nemolizumab significantly improved itch outcomes, disease severity, and quality of life measures from weeks 4 through 16 [55]. Collectively, from clinical trials on both AD and PN, itch reduction measures with receptor blockade preceded lesion improvement.

OSMRβ targeted therapies: More recently, clinical trials evaluating the clinical safety and efficacy of vixarelimab, a fully human monoclonal antibody targeting OSMRβ, have come to light. In a phase 2a clinical trial, vixarelimab treatment led to rapid and sustained improvements in the WI-NRS, with large-magnitude reductions observed as early as week three [56]. Higher rates of lesion clearance were also reported by week eight, and quality-of-life measures, including sleep disturbance, had significant improvements in early weeks of the trial. These findings were further supported by a phase 2b clinical trial that evaluated three different doses of vixarelimab compared to placebo. All doses of vixarelimab led to significant reductions in the WI-NRS compared to placebo, with higher doses producing a more significant effect [57]. Significant changes in pruritus measures were seen as early as week one and sustained through the rest of the trial course. Lesional clearance was also reported across all doses of vixarelimab compared to placebo.

Discussion

Inflammatory cytokines IL-31 and OSM are both implicated in the pathogenesis of itch, functioning as neuroimmune signaling cytokines that integrate immune activation, keratinocyte responses, and sensory neurons that drive chronic itch. While the mechanistic action of each is different, with IL-31 acting as a direct pruritogenic signal and OSM acting as a modulator of neuronal itch sensitization, studies showed that both cytokines serve an important role. Multiple cutaneous pruritic dermatoses have been studied in relation to these cytokines. AD and PN were among the most common pruritic cutaneous diseases assessed, with fewer studies directed towards psoriasis, FPLCA, dermatomyositis, and CTCL. Molecular targets most often studied were interactions between IL-31, IL-31RA, OSM, and OSMRβ. Overall, IL-31 protein expression was found to be increased within dermal inflammatory infiltrates across all pruritic dermatoses when compared to healthy controls; however, in some pruritic conditions, these levels were less positively correlated to itch.

The studies identified in this review highlight commonalities within the inflammatory pathway, supporting the idea that interactions of IL-31 and OSM correlate to itch. However, discrepancies between pruritic dermatoses were found. One study found induction of OSM to upregulate both STAT3 and STAT1 expression [47]. In contrast, another study found that OSM induced only STAT3 expression while downregulating STAT1 expression [46]. Furthermore, both IL-31 and IL-31RA were upregulated in epidermal keratinocytes of CTCL [33]. However, only IL-31 expression was upregulated in patients with AD [32]. While not all pruritic conditions were assessed, these findings suggest mechanistic differences dependent upon the specific disease. To better identify the downstream effects of IL-31 and OSM stimulation, more controlled studies are needed.

Previous studies have been able to establish causality; however, these studies are limited to non-human subjects, using mice for in vivo application. One study conducted by Feld et al. was able to link IL-31 to neuroplastic changes underlying chronic pruritus. This in vitro and in vivo study discovered that IL-31 acts on sensory neurons through the IL-31RA-STAT3 signaling, promoting nerve fiber outgrowth and branching [58]. Another study demonstrated that, in a non-human primate model, blockade of OSMRβ with a monoclonal antibody was found to attenuate IL-31 induced itch behavior, providing support for the shared IL-31/OSM receptor axis [59].

Other studies have been successful in providing clear mechanistic insight, specifically regarding OSM, OSMRβ, and their downstream effects. OSM has been found to directly activate nociceptive signaling in human dorsal root ganglion, a key substrate for itch and pain transmission [60]. In this study, activation of OSMRβ encouraged intracellular signaling via the MAP kinase-interacting serine/threonine kinase - eukaryotic translation initiation factor 4E (MNK-eIF4E) pathway, supporting a neuroimmune mechanism relevant to chronic pruritus and sensory hypersensitivity [60]. Similarly, OSM was found to activate STAT3 signaling in keratinocytes to induce IL-33, a key epithelial alarmin driving Th2 inflammation and pruritus [61]. Further supporting a neuroimmune itch framework in dermatologic conditions, a study evaluating the OSMRβ axis in PN found that lesional PN skin biopsies demonstrated significant increases of IL-31, IL-31RA, OSM, and OSMRβ protein in immune cells, epidermal cells, dermal nerves, and adnexal structures [62]. Though few studies provide deeper insight into the direct mechanistic actions of OSM, these findings nevertheless strengthen the neuroimmune-epithelial framework relevant to inflammatory pruritic dermatoses.

Clinical trials with targeted immunologic therapies, nemolizumab and vixarelimab, provided validation for the pathogenic roles of IL-31 and OSM cytokines. Studies found high clinical efficacy and safety profiles when utilized for the treatment of itch. These drugs work by targeting IL-31RA and OSMRβ, respectively. Blockage led to downregulation of inflammatory signaling and decreased pruritus. Though neuronal sensitization and activation are mechanisms exhibited by these cytokines, current human clinical trials lack the ability to establish causality. Previous studies utilizing mouse models have been successful in showing that blockade of OSMRβ in AD reduces pruritus, skin inflammation, Th2 cytokines, and dorsal root ganglion neuronal involvement [63]. Despite the impressive clinical improvements, further investigation of the neuroimmune effects in human subjects is necessary.

Limitations

While the included studies provided important details of both OSM and IL-31 signaling pathways, several limitations were present. The mechanistic, biopsy-based, and rare disease genetic studies had small sample sizes, restricting their statistical power and generalizability. Temporal relationships were not assessed in observational studies due to a lack of longitudinal evidence. Heterogeneity in patient populations, disease severity, and tissue sampling methods across studies may have contributed to different levels of cytokine and receptor expression. While the randomized controlled trials provided strong clinical evidence, most were limited by short treatment durations relative to the chronic disease being assessed, as well as demographic homogeneity, which limited generalizability to broader contexts. Many open-label extension studies also lack placebo-controlled groups.

Moreover, in our search of the current literature, a substantial portion of OSM studies were conducted on murine models and preclinical trials, leaving human in vitro studies as a large portion of supporting data for this cytokine. The in vitro designs were artificially stimulating healthy human-derived cells rather than lesional primary cells, which may not fully replicate in vivo conditions. Although converging molecular, translational, and interventional evidence strongly supported a neuroimmune framework for IL-31 and OSM signaling in chronic pruritus, there remains a lack of direct human neuronal functional and longitudinal studies. Future studies should prioritize functional validation through human sensory neurons, especially human DRGs. Clinical trials should evaluate IL-31RA and OSMRβ blockade in additional chronic pruritic dermatoses beyond AD and PN.

Our review highlights the complementary roles of IL-31 and OSM that drive itch, providing deeper insight into the mechanistic pathway promoted by activation of these cytokines. By mapping current literature, we provide a guide to potential therapeutic directions for chronic pruritic dermatoses.

## Conclusions

This systematic review identifies IL-31 and OSM as complementary neuroimmune cytokines involved in the pathogenesis of chronic pruritic dermatoses. Originating from activated immune cells, IL-31 and OSM bind to their corresponding receptor complexes containing the OSMRβ subunit, which are expressed on target cells, including keratinocytes and sensory neurons. This interaction leads to downstream activation of JAK1-driven STAT3 signaling, promoting epidermal barrier dysfunction, sustained inflammation, and persistent neuroimmune sensitization. For patients with AD and PN, inhibition of IL-31RA with nemolizumab and dual inhibition of OSMRβ with vixarelimab have demonstrated significant improvements in itch severity, patient-reported quality of life, and sleep disturbance. Given the essential role of JAK-STAT signaling within both pathways, these findings also support the therapeutic role of JAK inhibition in chronic pruritic dermatoses. Collectively, shared components of the IL-31 and OSM pathways, particularly OSMRβ and JAK1-STAT3, help understand itch pathogenesis in chronic pruritic dermatoses. Future research should prioritize long-term studies across multiple pruritic diseases to help clarify this pathway and optimize pharmacological interventions.

## References

[1] Whang KA, Khanna R, Williams KA, Mahadevan V, Semenov Y, Kwatra SG (2021). Health-related QOL and economic burden of chronic pruritus. J Invest Dermatol.

[2] Ständer S, Weisshaar E, Mettang T (2007). Clinical classification of itch: a position paper of the International Forum for the Study of Itch. Acta Derm Venereol.

[3] Brooks SG, Yosipovitch G (2024). Unmet needs in treating itch: reaching beyond eczema. J Dermatolog Treat.

[4] Burshtein J, Burshtein A, Schlesinger T (2026). Prurigo nodularis and the pain cascade: understanding the pathogenesis and approach to management. J Clin Aesthet Dermatol.

[5] Nguyen C, Thompson J, Nguyen DA, Wong CM, Scheufele CJ, Carletti M, Weis SE (2024). Presentations of cutaneous disease in various skin pigmentations: chronic atopic dermatitis. HCA Healthc J Med.

[6] Ständer S, Luger T, Kim B (2024). Cutaneous components leading to pruritus, pain, and neurosensitivity in atopic dermatitis: a narrative review. Dermatol Ther (Heidelb).

[7] Garcovich S, Maurelli M, Gisondi P, Peris K, Yosipovitch G, Girolomoni G (2021). Pruritus as a distinctive feature of type 2 inflammation. Vaccines (Basel).

[8] Cianferoni A, Spergel J (2014). The importance of TSLP in allergic disease and its role as a potential therapeutic target. Expert Rev Clin Immunol.

[9] Mishra SK, Wheeler JJ, Pitake S (2020). Periostin activation of integrin receptors on sensory neurons induces allergic itch. Cell Rep.

[10] Liu Q, Weng HJ, Patel KN, Tang Z, Bai H, Steinhoff M, Dong X (2011). The distinct roles of two GPCRs, MrgprC11 and PAR2, in itch and hyperalgesia. Sci Signal.

[11] Wang F, Kim BS (2020). Itch: a paradigm of neuroimmune crosstalk. Immunity.

[12] Ruppenstein A, Limberg MM, Loser K, Kremer AE, Homey B, Raap U (2021). Involvement of neuro-immune interactions in pruritus with special focus on receptor expressions. Front Med (Lausanne).

[13] Mazzetto R, Miceli P, Tartaglia J, Ciolfi C, Sernicola A, Alaibac M (2024). Role of IL-4 and IL-13 in cutaneous T cell lymphoma. Life (Basel).

[14] Tanaka M, Miyajima A (2003). Oncostatin M, a multifunctional cytokine. Reviews of Physiology, Biochemistry and Pharmacology.

[15] Zhou Y, Stevis PE, Cao J (2024). Structures of complete extracellular assemblies of type I and type II oncostatin M receptor complexes. Nat Commun.

[16] Liao V, Cornman HL, Ma E, Kwatra SG (2024). Prurigo nodularis: new insights into pathogenesis and novel therapeutics. Br J Dermatol.

[17] Park M, Jang J, Na K (2026). Oncostatin M drives multifaceted immune pathogenesis in atopic dermatitis-like inflammation via neutrophil-dependent mechanisms. Genes Dis.

[18] Auguste P, Guillet C, Fourcin M, Olivier C, Veziers J, Pouplard-Barthelaix A, Gascan H (1997). Signaling of type II oncostatin M receptor. J Biol Chem.

[19] Li L, Li ZE, Mo YL (2024). Molecular and cellular pruritus mechanisms in the host skin. Exp Mol Pathol.

[20] Roh YS, Choi J, Sutaria N, Belzberg M, Kwatra MM, Kwatra SG (2021). IL-31 inhibition as a therapeutic approach for the management of chronic pruritic dermatoses. Drugs.

[21] Bağci IS, Ruzicka T (2018). IL-31: a new key player in dermatology and beyond. J Allergy Clin Immunol.

[22] Cevikbas F, Wang X, Akiyama T (2014). A sensory neuron-expressed IL-31 receptor mediates T helper cell-dependent itch: involvement of TRPV1 and TRPA1. J Allergy Clin Immunol.

[23] Zhang Q, Putheti P, Zhou Q, Liu Q, Gao W (2008). Structures and biological functions of IL-31 and IL-31 receptors. Cytokine Growth Factor Rev.

[24] Furue M, Furue M (2021). Interleukin-31 and pruritic skin. J Clin Med.

[25] Huang IH, Chung WH, Wu PC, Chen CB (2022). JAK-STAT signaling pathway in the pathogenesis of atopic dermatitis: an updated review. Front Immunol.

[26] Oetjen LK, Mack MR, Feng J (2017). Sensory neurons co-opt classical immune signaling pathways to mediate chronic itch. Cell.

[27] Guttman-Yassky E, Irvine AD, Brunner PM (2023). The role of Janus kinase signaling in the pathology of atopic dermatitis. J Allergy Clin Immunol.

[28] Takahashi S, Ochiai S, Jin J (2023). Sensory neuronal STAT3 is critical for IL-31 receptor expression and inflammatory itch. Cell Rep.

[29] Page MJ, McKenzie JE, Bossuyt PM (2021). The PRISMA 2020 statement: an updated guideline for reporting systematic reviews. BMJ.

[30] Tufanaru C, Munn Z, Aromataris E, Campbell J, Hopp L (2025). Systematic reviews of effectiveness. JBI Manual for Evidence Synthesis - 2024 Edition.

[31] Sterne JA, Savović J, Page MJ (2019). RoB 2: a revised tool for assessing risk of bias in randomised trials. BMJ.

[32] Nobbe S, Dziunycz P, Mühleisen B, Bilsborough J, Dillon SR, French LE, Hofbauer GF (2012). IL-31 expression by inflammatory cells is preferentially elevated in atopic dermatitis. Acta Derm Venereol.

[33] Nattkemper LA, Martinez-Escala ME, Gelman AB, Singer EM, Rook AH, Guitart J, Yosipovitch G (2016). Cutaneous T-cell lymphoma and pruritus: the expression of IL-31 and its receptors in the skin. Acta Derm Venereol.

[34] Kim HJ, Zeidi M, Bonciani D, Pena SM, Tiao J, Sahu S, Werth VP (2018). Itch in dermatomyositis: the role of increased skin interleukin-31. Br J Dermatol.

[35] Boniface K, Diveu C, Morel F (2007). Oncostatin M secreted by skin infiltrating T lymphocytes is a potent keratinocyte activator involved in skin inflammation. J Immunol.

[36] Tseng PY, Hoon MA (2021). Oncostatin M can sensitize sensory neurons in inflammatory pruritus. Sci Transl Med.

[37] Suehiro M, Numata T, Saito R (2023). Oncostatin M suppresses IL31RA expression in dorsal root ganglia and interleukin-31-induced itching. Front Immunol.

[38] Sonkoly E, Muller A, Lauerma AI (2006). IL-31: a new link between T cells and pruritus in atopic skin inflammation. J Allergy Clin Immunol.

[39] Tey HL, Cao T, Nattkemper LA, Tan VW, Pramono ZA, Yosipovitch G (2016). Pathophysiology of pruritus in primary localized cutaneous amyloidosis. Br J Dermatol.

[40] Dai X, Shiraishi K, Muto J, Utsunomiya R, Mori H, Murakami M, Sayama K (2022). Nuclear IL-33 plays an important role in IL-31-mediated downregulation of FLG, keratin 1, and keratin 10 by regulating signal transducer and activator of transcription 3 activation in human keratinocytes. J Invest Dermatol.

[41] Lee CH, Hong CH, Yu WT (2012). Mechanistic correlations between two itch biomarkers, cytokine interleukin-31 and neuropeptide β-endorphin, via STAT3/calcium axis in atopic dermatitis. Br J Dermatol.

[42] Kasraie S, Niebuhr M, Baumert K, Werfel T (2011). Functional effects of interleukin 31 in human primary keratinocytes. Allergy.

[43] Deng J, Liao V, Parthasarathy V (2023). Modulation of neuroimmune and epithelial dysregulation in patients with moderate to severe prurigo nodularis treated with nemolizumab. JAMA Dermatol.

[44] Wang H, Lei L, Hu J, Li Y (2020). Oncostatin M upregulates Livin to promote keratinocyte proliferation and survival via ERK and STAT3 signalling pathways. Exp Physiol.

[45] Kanda N, Watanabe S (2012). Increased serum human β-defensin-2 levels in atopic dermatitis: relationship to IL-22 and oncostatin M. Immunobiology.

[46] Gazel A, Rosdy M, Bertino B, Tornier C, Sahuc F, Blumenberg M (2006). A characteristic subset of psoriasis-associated genes is induced by oncostatin-M in reconstituted epidermis. J Invest Dermatol.

[47] Richards CD, Gandhi R, Botelho F, Ho L, Paolini JF (2020). Oncostatin M induction of monocyte chemoattractant protein 1 is inhibited by anti-oncostatin M receptor beta monoclonal antibody KPL-716. Acta Derm Venereol.

[48] Hashimoto T, Nattkemper LA, Kim HS (2021). Itch intensity in prurigo nodularis is closely related to dermal interleukin-31, oncostatin M, IL-31 receptor alpha and oncostatin M receptor beta. Exp Dermatol.

[49] Arita K, South AP, Hans-Filho G (2008). Oncostatin M receptor-β mutations underlie familial primary localized cutaneous amyloidosis. Am J Hum Genet.

[50] Saeedi M, Ebrahim-Habibi A, Haghighi A, Zarrabi F, Amoli MM, Robati RM (2014). A novel missense mutation in oncostatin M receptor beta causing primary localized cutaneous amyloidosis. Biomed Res Int.

[51] Ruzicka T, Hanifin JM, Furue M (2017). Anti-interleukin-31 receptor A antibody for atopic dermatitis. N Engl J Med.

[52] Silverberg JI, Pinter A, Pulka G (2020). Phase 2B randomized study of nemolizumab in adults with moderate-to-severe atopic dermatitis and severe pruritus. J Allergy Clin Immunol.

[53] Silverberg JI, Wollenberg A, Reich A (2024). Nemolizumab with concomitant topical therapy in adolescents and adults with moderate-to-severe atopic dermatitis (ARCADIA 1 and ARCADIA 2): results from two replicate, double-blind, randomised controlled phase 3 trials. Lancet.

[54] Ständer S, Yosipovitch G, Legat FJ (2025). Efficacy and safety of nemolizumab in patients with moderate to severe prurigo nodularis: the OLYMPIA 1 randomized clinical phase 3 trial. JAMA Dermatol.

[55] Kwatra SG, Yosipovitch G, Legat FJ (2023). Phase 3 trial of nemolizumab in patients with prurigo nodularis. N Engl J Med.

[56] Sofen H, Bissonnette R, Yosipovitch G (2023). Efficacy and safety of vixarelimab, a human monoclonal oncostatin M receptor β antibody, in moderate-to-severe prurigo nodularis: a randomised, double-blind, placebo-controlled, phase 2a study. EClinicalMedicine.

[57] Ständer S, Yosipovitch G, Sofen H, Abramzon D, Galanter J, Wang S, Paolini JF (2026). Vixarelimab in patients with prurigo nodularis: a randomized clinical trial. JAMA Dermatol.

[58] Feld M, Garcia R, Buddenkotte J (2016). The pruritus- and TH2-associated cytokine IL-31 promotes growth of sensory nerves. J Allergy Clin Immunol.

[59] Gandhi R, Crowder K, Barrow K, Paolini JF (2019). KPL-716, an anti-oncostatin M receptor β (OSMRβ) monoclonal antibody, reduces IL-31-induced scratching behavior in cynomolgus monkeys: establishment and optimization of a PK/PD model. J Invest Dermatol.

[60] Mwirigi J, Franco-Enzastiga UM, Sankaranarayanan I (2023). Oncostatin M induces nociceptive signaling in human dorsal root ganglia. J Pain.

[61] Tsuda H, Komine M, Tominaga S, Ohtsuki M (2015). Oncostatin M induces IL-33 through STAT3 pathway in normal human epidermal keratinocytes. J Invest Dermatol.

[62] Mikhak Z, Ständer S, Metze D (2019). The oncostatin M receptor beta axis identified in prurigo nodularis. J Invest Dermatol.

[63] Komori T, Hisaoka T, Kotaki A, Iwamoto M, Miyajima A, Esashi E, Morikawa Y (2024). Blockade of OSMRβ signaling ameliorates skin lesions in a mouse model of human atopic dermatitis. FASEB J.

